# Association of Systemic Hormonal Contraceptives With Reconstruction Procedures for Patellar Instability

**DOI:** 10.1177/23259671231191786

**Published:** 2023-10-04

**Authors:** Brandon J. Martinazzi, Nicholas Bertha, Hannah H. Nam, F. Jeffrey Lorenz, Vincenzo Bonaddio, Anna Ptasinski, Robert A. Gallo

**Affiliations:** † Department of Orthopaedics and Rehabilitation, Penn State Health, Milton S. Hershey Medical Center, Hershey, Pennsylvania, USA.; *Investigation performed at Penn State Health, Milton S. Hershey Medical Center, Hershey, Pennsylvania, USA*

**Keywords:** estrogen, female athlete, ligamentous laxity, patellar instability, progesterone

## Abstract

**Background:**

Previous research suggests that estrogen plays a role in increased ligamentous laxity observed within the female population. Whereas many studies have sought to evaluate the impact of exogenous estrogen on anterior cruciate ligament injuries, research has not yet explored its impact on the medial patellofemoral ligament. Furthermore, less is known about the role of exogenous progesterone on ligamentous structures.

**Purpose:**

To determine whether women prescribed systemic estrogen (ethinyl estradiol) or progesterone (norgestimate or etonogestrel) hormonal contraceptives had an increased risk of undergoing reconstruction surgery for patellar instability compared with women without a prescription for systemic hormonal contraceptives.

**Study Design:**

Cross-sectional study; Level of evidence, 3.

**Methods:**

The TriNetX Research Network database was queried using International Classification of Disease and Common Procedural Terminology codes for women aged 15 to 26 years who underwent reconstruction procedures for patellar instability between 2012 and 2022. Women were grouped according to whether they had a coded prescription for a systemic hormonal contraceptive containing either ethinyl estradiol or etonogestrel; controls were matched by age, sex, race, and ethnicity. The relative risk (RR) of undergoing reconstruction for patellar instability was determined for each contraceptive.

**Results:**

After 1-to-1 propensity score matching, 0.054% (525/980,878) of women prescribed a systemic contraceptive containing ethinyl estradiol underwent reconstruction procedures for patellar instability compared with 0.043% (417/980,878) of controls (RR, 1.3; 95% CI, 1.1-1.4; *P* = .0004). Likewise, 0.058% (67/116,260) of women prescribed a form of systemic contraceptive containing only etonogestrel underwent reconstruction procedures for patellar instability compared with 0.026% (30/116,260) of controls (RR, 2.2; 95% CI, 1.5-3.4; *P* = .0002).

**Conclusion:**

Female patients prescribed systemic contraceptives containing estrogen or progesterone had an increased rate of reconstruction procedures for patellar instability.

Anterior cruciate ligament (ACL) injuries are common among female athletes.^1^ Many believe female anatomy, neuromuscular control, and ligamentous laxity contribute to the high incidence of ACL injury.^9^ Furthermore, some posit that estrogen mediates the increased ligamentous laxity seen among the female population.^3^ However, whether differences in estrogen levels increase or decrease the risk of ACL injury remains controversial.^8,14^ A study by Rahr-Wagner et al^18^ suggests oral contraceptives actually provide a protective effect. Data from the Centers for Disease Control and Prevention states that, from 2017 to 2019, 24.4% of women aged 15 to 49 years utilized hormonal contraceptives, with approximately half of those patients using long-acting reversible contraceptives.^6^ Therefore, there is growing interest among researchers to determine whether contraceptives contribute to ligamentous injury about the knee.

Persistent patellar instability is more common in the female population.^7^ There are multiple factors that may contribute to this disparity. For example, male athletes have a greater condylar height and width compared with their female counterparts.^10^ Likewise, women have greater rotation of the tibia, as well as a greater Q angle, which may place added stress on ligaments about the patella, predisposing them to persistent instability.^15,20,23^ Furthermore, increased ligamentous laxity in women may increase the risk of developing persistent patellar instability.^11^

Given that estrogen has an effect on various connective tissues including bone,^5^ muscles,^21^ cartilage,^2^ it is plausible that higher levels of estrogen seen in the female patients compared with the male population may predispose them to persistent patellar instability. Likewise, progesterone receptors have been found on various connective tissues, and progesterone also likely plays a role in the normal structure and function of tissue.^12,13^ Wojtys et al^22^ stated that knee injuries are more common during the ovulation phase. It is believed that this is related to the luteinizing hormone spike, which is associated with higher estrogen levels. This spike subsequently leads to upregulation of relaxin, which increases ligamentous laxity.^22^

While the effect of estrogen has been studied most extensively in ACL injuries, there is a paucity of research exploring its role in other ligamentous injuries about the knee. Moreover, even less is known about the role of progesterone. Therefore, the purpose of this study was to determine whether female patients prescribed systemic hormonal estrogen or progesterone contraception had an increased risk of persistent patellar instability requiring surgical intervention. We hypothesized that female patients prescribed estrogen or progesterone contraceptives would have an increased risk of persistent patellar instability requiring a reconstructive procedure compared with those not prescribed systemic contraceptives.

## Methods

Data were collected from the TriNetX Research Network. This network includes electronic medical records from over 100 million persons and more than 50 health care organizations. All data processed within the TriNetX Network comply with the Health Insurance Portability and Accountability Act and contain only deidentified patient information. As such, this study was exempt from institutional review board approval.

The database was queried using International Classification of Disease, 10th Revision, and Common Procedural Terminology (CPT) codes, previously described by Moreland et al^16^ to identify women aged 15 to 26 years who underwent reconstruction procedures for patellar instability between 2012 and 2022 and who had a coded prescription for any form of systemic contraceptive (pills, implants, rings) that contained ethinyl estradiol (synthetic estrogen) or etonogestrel (progestin derivative) without ethinyl estradiol. The primary outcome of interest was the rate of patellar reconstruction procedures within 5 years after inception of the systemic contraceptive. Information detailing the contraceptives and CPT codes is reported in Tables 1 and 2, respectively.

**Table 1:** Description of Systemic Contraceptives Evaluated

Drug	Main Ingredient	Mechanism of Action	Examples
Ethinyl estradiol^21^	Synthetic estrogen	Acts on the hypothalamic gonadal axis to prevent ovulation	Main ingredient in many forms of oral contraceptive pills and some implants. Often combined with progestins
Etonogestrel^22^	Progestin derivative	Acts on the hypothalamic gonadal axis to prevent ovulation	Subdermal implant when used alone

**Table 2:** CPT and Medication Codes Utilized*^a^*

	Description
CPT code	
27427	Under repair, revision, and/or reconstruction procedures on the femur (thigh region) and knee joint
27424	Under repair, revision, and/or reconstruction procedures on the femur (thigh region) and knee joint
27422	Under repair, revision, and/or reconstruction procedures on the femur (thigh region) and knee joint
27420	Reconstruction of dislocating patella
Medication code	
4124	Ethinyl estradiol
14584	Etonogestrel

*^a^* CPT, Common Procedural Terminology.

After identification of our cohort, we conducted 1-to-1 propensity score matching by age, sex, race, and ethnicity with controls who did not have a coded prescription for a systemic hormonal contraceptive that contained ethinyl estradiol or etonogestrel. Demographic data from each cohort was collected (Table 3).

**Table 3:** Cohort Characteristics vs Controls for Ethinyl Estradiol and Etonogestrel*^a^*

	Ethinyl Estradiol					
	Before Matching			After Matching		
	Contraceptive (n = 980,878)	Control (n = 5,044,162)	*P*	Contraceptive (n = 980,878)	Control (n = 980,878)	*P*
Age, y	20.5 ± 3.4	20.1 ± 3.64	<.0001	20.5 ± 3.4		
Sex						
Male	0 (0%)	0 (0%)		0 (0%)	0 (0%)	
Female	980,878 (100%)	5,044,162 (100%)		980,878 (100%)	980,878 (100%)	
Race						
White	682,896 (70%)	2,716,118 (54%)	<.0001	682,896 (70%)	682,896 (70%)	>.99
Black or African American	142,097 (14%)	896,835 (18%)	<.0001	142,097 (14%)	142,099 (14%)	.99
Asian	23,282 (2%)	163,080 (3%)	<.0001	23,282 (2%)	23,282 (2%)	>.99
Native Hawaiian or other Pacific Islander	1,106 (<1%)	10,794 (<1%)	<.0001	1,106 (<1%)	1,104 (<1%)	.99
American Indian or Alaska native	2998 (<1%)	23,015 (<1%)	<.0001	2998 (< 1%)	2,998 (<1%)	>.99
Unknown	128,499 (13%)	1,234,320 (24%)	<.0001	128,499 (13%)	128,499 (13%)	>.99
Ethnicity						
Not Hispanic or Latino	750,062 (76%)	2,780,442 (55%)	<.0001	750,062 (76%)	750,062 (76%)	>.99
Hispanic or Latino	89,809 (9%)	590,608 (12%)	<.0001	89,809 (9%)	89,809 (9%)	>.99
Unknown	141,007 (15%)	1,673,112 (33%)	<.0001	141,007 (15%)	141,007 (15%)	>.99
	Etonogestrel					
	Before Matching			After Matching		
	Contraceptive (n = 116,260)	Control (n = 5,044,162)	*P*	Contraceptive (n = 116,260)	Control (n = 116,260)	*P*
Age, y	20.8 ± 3.3	20.1 ± 3.6	<.0001	20.8 ± 3.3	20.8 ± 3.3	>.99
Sex						
Male	0 (0%)	0 (0%)		0 (0%)	0 (0%)	
Female	116,260 (100%)	5,044,162 (100%)		116,260 (100%)	116,260 (100%)	
Race						
White	62,107 (53%)	2,716,118 (54%)	.004	62,107 (53%)	62,107 (53%)	>.99
Black or African American	27,802 (24%)	896,835 (18%)	<.0001	27,802 (24%)	27,802 (24%)	>.99
Asian	1918 (1.6%)	163,080 (3%)	<.0001	1918 (1.6%)	1918 (1.6%)	>.99
Native Hawaiian or other Pacific Islander	208 (<1%)	10,794 (<1%)	.01	208 (<1%)	208 (<1%)	>.99
American Indian or Alaska native	1285 (1%)	23,015 (<1%)	<.0001	1285 (1%)	1285 (1%)	>.99
Unknown	22,940 (20%)	1,234,320 (24%)	<.0001	22,940 (20%)	22,940 (20%)	>.99
Ethnicity						
Not Hispanic or Latino	71,834 (62%)	2,780,442 (55%)	<.0001	71,834 (62%)	71,834 (62%)	>.99
Hispanic or Latino	22,656 (19%)	590,608 (12%)	<.0001	22,656 (19%)	22,656 (19%)	>.99
Unknown	21,770 (19%)	1,673,112 (33%)	<.0001	21,770 (19%)	21,770 (19%)	>.99

*^a^* Sum of percentages may not be 100% due to rounding. Data are presented as number of patients and percentages.

All statistical analyses were performed within the TriNetX platform. Relative risk (RR) and associated 95% confidence intervals (CIs) were calculated to compare the rate of patellar reconstruction surgery in patients prescribed systemic contraceptives versus controls, both before and after propensity score matching. For the purposes of our study, statistical significance was defined as *P* < .05.

## Results

During the study period, 0.054% (525/980,878) of women prescribed a systemic contraceptive containing ethinyl estradiol underwent a reconstruction procedure for patellar instability, compared with 0.039% (1978/5,044,162) of controls. This difference was statistically significant (*P* < .0001; RR, 1.4; 95% CI, 1.2-1.5). Likewise, women with a prescription for a contraceptive containing etonogestrel without ethinyl estradiol were significantly more likely to undergo reconstruction procedures for patellar instability compared with unmatched controls (0.058% [67/116,260] vs 0.039% [1978/5,044,162], respectively; *P* = .0018, RR, 1.5; 95% CI, 1.2-1.9) (Table 4). Even after 1-to-1 propensity score matching by age, sex, race, and ethnicity, women with a prescription for either ethinyl estradiol (*P* = .0004; RR, 1.3; 95% CI, 1.1-1.4) or etonogestrel (*P* = .0002; RR, 2.2; 95% CI, 1.5-3.4) were more likely to undergo reconstruction procedures for patellar instability compared with controls (Table 4).

**Table 4:** Association Between Systemic OC and Patellar Reconstruction Procedures Before and After Propensity Score Matching*^a^*

	Cohort Size	Reconstruction Procedure (n)	Rate of Reconstruction	RR (95% CI)	*P*
Before matching					
Ethinyl estradiol				1.4 (1.2-1.5)	<.0001
Contraceptive	980,878	525	0.054%		
Control	5,044,162	1978	0.039%		
Etonogestrel				1.5 (1.2-1.9)	.0018
Contraceptive	116,260	67	0.058%		
Control	5,044,162	1978	0.039%		
After matching					
Ethinyl estradiol				1.3 (1.1-1.4)	.0004
Contraceptive	980,878	525	0.054%		
Control	980,878	417	0.043%		
Etonogestrel				2.2 (1.5-3.4)	.0002
Contraceptive	116,260	67	0.058%		
Control	116,260	30	0.026%		

*^a^* OC, oral contraceptive; RR, relative risk.

## Discussion

The study findings indicated that female patients who were prescribed a systemic hormonal contraceptive that contained either ethinyl estradiol or etonogestrel were significantly more likely to undergo subsequent reconstruction procedures for patellar instability compared with control patients. Further, this association persisted after matching for age, sex, race, and ethnicity. Therefore, we accept our study hypothesis that patients prescribed estrogen- or progesterone-containing systemic contraceptives would be more likely to undergo reconstruction procedures for patellar instability. Our study is the first to identify an association between systemic estrogen or progesterone-containing contraceptives and reconstruction procedures for patellar instability.

Despite differences in sex-based anatomy of the trochlea and patella, our study evaluated only a female population in an attempt to limit the effects of these differences. We hypothesize that increased estrogen from oral contraceptives supplementation may increase laxity about the knee. This laxity may also include internal rotation of the tibia, making these patients at a higher propensity for chronic instability.^20^

There are some limitations in the data collected. Some of these limitations stem from utilizing a large online database, which relies on accurate coding. Because of the use of the online database system, our sample only included patients who were part of a health system that uses the TriNetX research network. Furthermore, we were not able to determine the specific route of administration of contraceptive for each group. For example, our ethinyl estradiol group contained all patients on any form of contraceptive (pill, shot, implant, etc) that had ethinyl estradiol as the active ingredient. Thus, while we were able to identify an association between systemic estrogen and reconstruction procedures for patellar instability, we failed to capture what specific type of contraceptive method had the strongest association.

In addition, given that we cannot evaluate or follow the individual patient, we have to make the assumption that patients prescribed a form of systemic hormonal contraceptive were taking medication in the prescribed manner. Moreover, while we matched for age, sex, race, and ethnicity, we did not obtain information regarding activity level, nonoperative treatment methods utilized for each cohort, or other potential risk factors, such as an elevated Beighton score, which has previously been described as a potential risk factor for patellar instability.^4,17,19^ Another limitation is a lack of a code specific for medial patellofemoral ligament reconstruction, which has become the gold standard for remedying patellar instability. While code 27427 is often used to bill for this procedure, this code also encompasses other extra-articular ligament reconstructions, such as collateral ligament reconstructions. We show the other CPT codes listed in Appendix Table A1 to include all of the reconstruction procedures for patellar instability; however, the majority of procedures were coded as 27427.

Finally, it is important to consider the rarity of these procedures, as well as the absolute risk difference. For example, when looking at our etonogestrel group, which had an RR of approximately 2, the absolute risk difference would be 0.04%. Using this value, one can determine that the number needed to harm, or the number of patients that would need to be taking a systemic contraceptive for 1 additional patient to experience an adverse outcome (reconstruction procedure for patellar instability) would be 2500. In general, a larger number needed to harm is beneficial; however, one may ask how high is acceptable? As such, we are certainly not suggesting that physicians counsel young women to stop using systemic contraceptives. Rather, our data simply identify a new association and will hopefully inspire further research to determine whether hormones like estrogen and progesterone may contribute to recurrent patellar instability in the female population.

Despite these limitations, we feel that our findings are important as they explore the role of estrogen and progesterone on other ligamentous stabilizers about the knee. We hope this study contributes to future research that will help elucidate the role of estrogen and progesterone in patellar instability. Furthermore, if future research does determine that estrogen and progesterone contraceptives contribute to patellar instability, we can work to develop early interventions for patients on these medications to prevent the need for surgical intervention.

## Conclusion

Female patients prescribed estrogen- or progesterone-containing systemic contraceptives had an increased rate of reconstruction procedures for patellar instability. We hypothesize that this association may be the result of the hormonal effects on laxity of the soft tissues; however, further research is needed to help determine the underlying pathophysiology of this phenomenon.

## Appendix

**Table A1:** Breakdown of Number of Procedures Before Matching According to CPT Code*^a^*

CPT Code	Ethinyl Estradiol^b^	Control^b^
27427	334	1320
27424	<10	15
27422	141	540
27420	93	345
CPT Code	Etonogestrel	Control
27427	49	1320
27424	<10	15
27422	14	540
27420	11	345

*^a^* CPT, Common Procedural Terminology.

*^b^* Number of patients with that particular code identified in the database.
